# Collective cell dynamics and luminal fluid flow in the epididymis: A mechanobiological perspective

**DOI:** 10.1111/andr.13490

**Published:** 2023-07-17

**Authors:** Veronica Lee, Barry T. Hinton, Tsuyoshi Hirashima

**Affiliations:** 1 Mechanobiology, Institute, National University of Singapore, Singapore, Singapore; 2 Department of Cell Biology, University of Virginia School of Medicine, Charlottesville, Virginia, USA; 3 Department of Physiology, Yong Loo Lin School of Medicine, National University of Singapore, Singapore, Singapore

**Keywords:** collective cell dynamics, epididymis, luminal fluid flow, mechanobiology, Wolffian duct

## Abstract

**Background:**

The mammalian epididymis is a specialized duct system that serves a critical role in sperm maturation and storage. Its distinctive, highly coiled tissue morphology provides a unique opportunity to investigate the link between form and function in reproductive biology. Although recent genetic studies have identified key genes and signaling pathways involved in the development and physiological functions of the epididymis, there has been limited discussion about the underlying dynamic and mechanical processes that govern these phenomena.

**Aims:**

In this review, we aim to address this gap by examining two key aspects of the epididymis across its developmental and physiological phases.

**Results and Discussion:**

First, we discuss how the complex morphology of the Wolffian/epididymal duct emerges through collective cell dynamics, including duct elongation, cell proliferation, and arrangement during embryonic development. Second, we highlight dynamic aspects of luminal fluid flow in the epididymis, essential for regulating the microenvironment for sperm maturation and motility, and discuss how this phenomenon emerges and interplays with epididymal epithelial cells.

**Conclusion:**

This review not only aims to summarize current knowledge but also to provide a starting point for further exploration of mechanobiological aspects related to the cellular and extracellular fluid dynamics in the epididymis.

## 1 | INTRODUCTION

The epididymis is a vital organ in the mammalian male reproductive system, responsible for the storage and maturation of spermatozoa.^1,2^ This complex organ exhibits a unique morphology, consisting of a highly coiled and convoluted tubular structure that is divided into several interconnected segments with distinct anatomical and functional properties. This intricate tissue architecture offers an opportunity to investigate the relationship between morphology and reproductive function, including male fertility and reproductive health, and understanding the mechanical and dynamic properties of the epididymis is crucial for deciphering this link. Recent advances in imaging, genetics, and biophysics have enabled the exploration of the mechanical and dynamic properties of the epididymis at the cellular and molecular level, providing new insights into the mechanisms of developmental and physiological aspects.

This short review examines the dynamic and mechanical features of tissues, cells, and molecules in the epididymis that arise during embryonic development and mature stages, drawing insights from earlier experiments conducted on mice and rats. We first highlight the cellular processes underlying the elongation, bending, and folding morphogenesis in the developing epididymis, with a particular focus on cell migration, proliferation, and rearrangement. We then discuss the dynamic properties of luminal hydraulics within the epididymal duct lumen and their potential impact on the physiological function of the epididymis.

## 2 | COLLECTIVE CELL DYNAMICS IN THE DEVELOPING WOLFFIAN/EPIDIDYMAL DUCT

Collective cell dynamics refers to the phenomenon in which individual cells work together to create a coordinated behavior. In embryonic development, the Wolffian duct or epididymal duct, which serves as the primordia of the epididymis, undergoes a complex series of morphogenetic events, culminating in the formation of the characteristic tubular coiling pattern. These morphogenetic events are orchestrated by collective cell dynamics, which consist of cell migration, proliferation, and rearrangement, each of which is essential for the proper formation and organization of the epididymal structure. We first discuss the cellular mechanisms that underlie the elongation of the Wolffian duct during the initial phase of male reproductive tract development. We then proceed to discuss how distinct morphological modes of the epididymis, such as folding and coiling, are achieved from a viewpoint of dynamics and mechanics. Lastly, we explore the cellular mechano-response system that serves as the foundation for maintaining the radial size of the developing structure.

### 2.1 | Elongation of the duct in the early phase

During vertebrate embryonic development, the mesodermal layer, one of the primary germ layers, differentiates into three distinct segments: the paraxial, intermediate, and lateral plate mesoderm. Among these regions, the intermediate mesoderm gives rise to the Wolffian duct or mesonephric duct, which serves as progenitors of the male reproductive tracts, including the epididymis and vas deferens.^3^ Malformation of the Wolffian ducts often results in the congenital anomalies, such as the formation of duplex ureters and the absence of the vas deferens,^4^ making an understanding of the basic developmental processes critically important. Although androgens largely regulate the development of the Wolffian ducts into male internal genitalia, the proper formation of the male reproductive tract can only be achieved with the correct spatial organization of its cells, which depends on complex interplay of cell signaling and cell dynamics in the epithelium and mesenchyme.^5,6^

In the intermediate mesoderm, cells at the posterior end collectively migrate toward the cloaca, the primordium of the bladder and urethra, whereas a specialized subset of cells undergoes a process of fate specification in the anterior region. This collective cell migration of the Wolffian duct is mediated by a gradient of fibroblast growth factor 8 (FGF8) as demonstrated in avian development.^7^ The concentration of FGF8 is highest at the posterior end and lowest at the anterior end. As the duct elongates posteriorly, the gradient also moves along the anterior–posterior axis in the same direction of the elongation, and the static cells in the anterior region, which receive lower levels of FGF8, undergo epithelialization. Thus, in this context, FGF8 acts as a chemoattractant to the leading cells of the elongating Wolffian duct and prevents it from undergoing epithelialization. These findings suggest that an FGF8 gradient plays a role in coordinating the elongation of the Wolffian duct and maintaining its epithelial integrity.^8^ However, it has been proposed that FGF signaling provides motility for the collective migration, instead of directing the migration path of the duct, as evidenced by the fact that a localized source of FGF8 placed adjacent to the duct did not affect its migration path.^9^ Further investigations are required to clarify the roles of FGF signaling in the collective cell migration during the elongation of the Wolffian duct.

The initiation of collective cellular migration is concomitant with the induction of hydrodynamic flow, resulting from the motility of cilia in the zebrafish pronephros, which originates from the intermediate mesoderm. It has been postulated that the movement of fluid in the lumen is a prerequisite for the occurrence of collective cellular migration.^10^ Moreover, the collective cellular migration induced by fluid flow results in cellular elongation, which in turn triggers the PI3K signaling cascade, leading to enhanced cellular proliferation. Blocking cell proliferation by inhibiting PI3K activity results in premature termination of cellular migration and can cause cellular overstretching, which points toward the existence of a synergistic relationship between flow-mediated cellular migration and cellular proliferation during tissue elongation.^11^ There has been increasing interest in elucidating the role of luminal hydraulics in tissue morphogenesis of developing organs, encompassing phenomena, such as fluid flow and hydrostatic pressure.^12–14^ Despite these promising phenomenological reports, the mechanism by which the epithelial duct cells can perceive and respond to the luminal fluid flow is not yet fully understood.

### 2.2 | Bending and coiling of the duct

**FIGURE 1:**
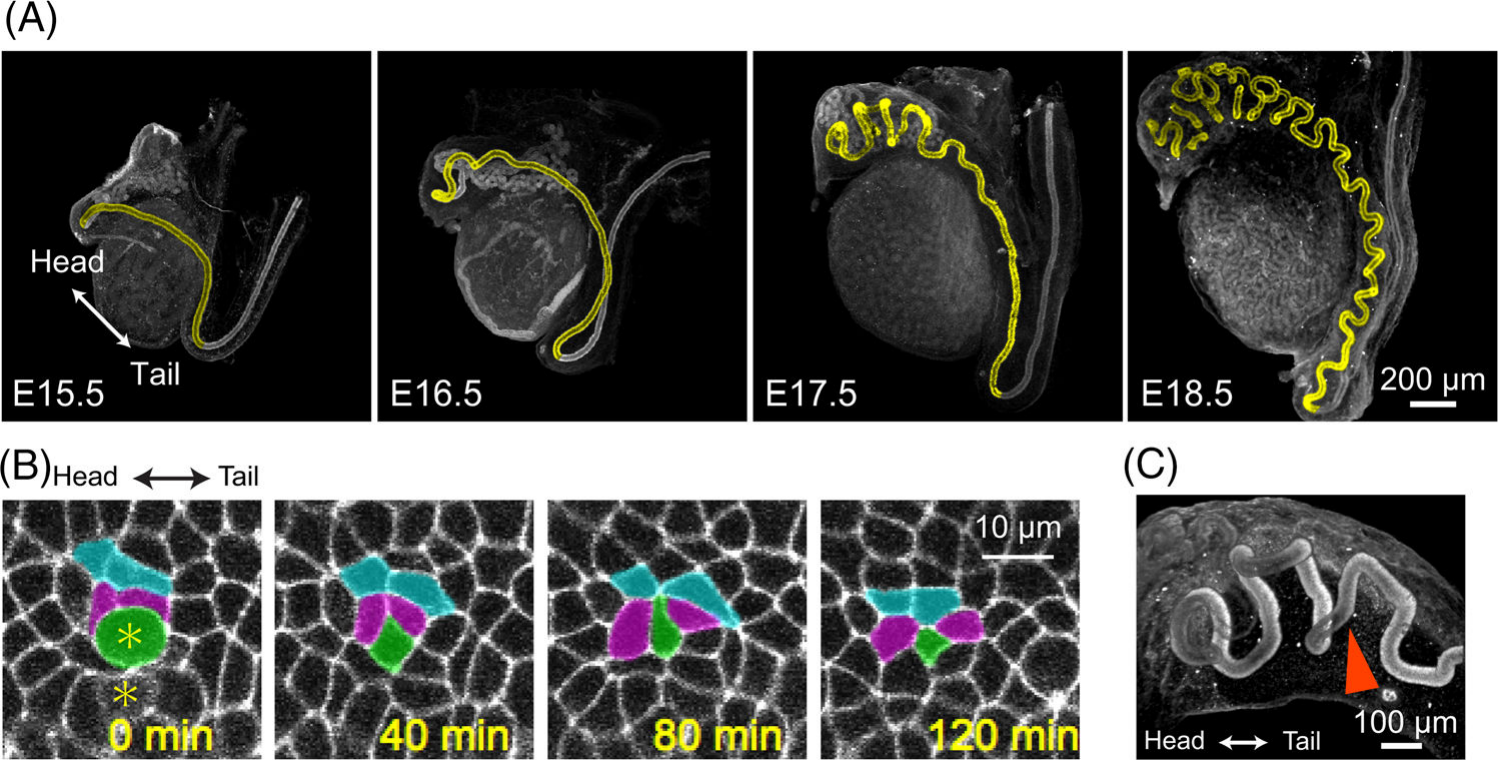
Morphogenetic events in the murine Wolffian duct. (A) Developmental process of murine Wolffian duct, depicted in yellow, from E15.5 to E18.5. Scale bar, 200 *μ*m. The images were reproduced with slight modifications from a previous publication^15^ under the CC BY 3.0 license. (B) Time-lapse snapshots of epithelial cells in a developing murine Wolffian duct. The two daughter cells resulting from cell division are denoted by yellow asterisks, with one of the daughter cells highlighted in green. A group of cells, including the green cell and four adjacent cells, colored in magenta and light blue undergo cell intercalation. Scale bar, 10 *μ*m. The images were reproduced from a previous publication^22^ under the CC BY 4.0 license. (C) A three-dimensional view of the duct, where the arrowhead indicates a perversion, where the helical handedness of the duct changes. Scale bar, 100 *μ*m. The image was reproduced from a previous publication^15^ under the CC BY 3.0 license.

Development of Wolffian duct occurs in both male and female embryos, but the duct is sustained solely in males via the influence of testosterone and eventually gives rise to a part of the male reproductive tract, including the epididymis. In mice, at embryonic day (E) 14.5, the epididymal duct appears relatively straight, and it begins to bend and fold in the anterior (head) region at E15.5–E16.5. The duct subsequently progresses to coiling, leading to the formation of a highly intricate, convoluted three-dimensional structure in the anterior region at E17.5, followed by the folding in the posterior (tail) region at E18.5^15,16^ (Figure 1A). Notably, this sequence of elaborate morphogenesis is accomplished via the coordinated coupling of relatively simple regimes of cellular and signaling processes regulation, rendering it highly stereotypical in nature.^17^ The most critical tissue event for this morphogenetic process is duct elongation as described below.

The elongation of the Wolffian duct results from mainly two factors: cell proliferation and cell intercalation. Cell proliferation is a primary driver for the increase in the volume of the epithelial duct and is controlled by inhibin beta A (Inhba), a subunit of TGF-beta super-family proteins, in the developing murine epididymis.^18^ Importantly, *Inhba* expresses locally in the anterior region of the Wolffian duct mesenchyme at E15.5–E17.5 and in the posterior region as well at E18.5, indicating a spatial correlation between the expression of *Inhba* and the morphogenetic processes of tissue bending and folding.^15,18^ Cell intercalation or rearrangement is another essential collective cell behavior that drives duct elongation. This process involves the mechanical stress by cells on neighboring cells to reshape the tissue by changing their positions relative to each other.^19,20^ In the Wolffian duct, the epithelial cells undergo mediolateral cell intercalation or convergent extension, which is regulated by protein tyrosine kinase 7 (Ptk7).^21^ In the developing murine Wolffian duct, a multicellular process, known as T1 transition (or topological rearrangement process of the first kind), has been identified, where two pairs of cells swap their neighbors in a confluent cellular monolayer, driving duct elongation^21,22^ (Figure 1B). The contacts between adjacent cells differ in their apical and basal regions, initially giving the impression that the cells have twisted around each other. Recent reports have identified a unique cell shape called a scutoid, which resembles the cell arrangement found within the Wolffian duct, suggesting that scutoids would be present within them.^23–25^ Presumably, this unique cell shape allows for efficient cell intercalation and provides stability for the curved tubular epithelial tissues during elongation.

Elongation rate of murine Wolffian duct increases over time during the developmental phase,^15^ and the epididymal duct attains a length of approximately 0.8 m in adulthood.^26^ To accommodate the extreme length of the duct to fit within the confines of the scrotum, the elongating duct must undergo highly organized coiling to be efficiently packed within the finite volume. This coiling morphogenesis of the duct takes place in a series of events coupling between mechanical and signaling effects. For example, the induction of cell proliferation by Inhba in the anterior region results in strong compressive forces across the region of the epithelial duct, leading to mechanical buckling of the tissue, which is one of the structural instability phenomena.^27^ As a consequence, the relatively straight duct becomes unstable and develops large bends, with the initial shape of the duct, its wavelength, and amplitude being spontaneously determined mechanically, whereas the global positions of bending and folding in the epididymis are genetically pre-determined during development.^15^ Despite gaining mechanical insight into the bending process, it is still unclear how the coiling process occurs and what factors determine its shape. Importantly, careful observation at E17.5 reveals the existence of perversion where the direction of rotation of the coiling duct reverses^15^ (Figure 1C), suggesting that a unidirectional torsion or mechanical forces that twist the Wolffian duct are unlikely to generate the force responsible for the shape of the duct. Furthermore, the formation of specific septa, which partitions the duct into discrete regions, occurs concurrently with the progress of duct folding and is a critical process for sculpting the characteristic coiling shape.^17^ The mechanisms by which they occur and how specific regions are selected are unknown, thus requiring further investigation.

### 2.3 | Radial size maintenance of the growing duct

Both cellular proliferation and intercalation play a significant role in the longitudinal growth of developing Wolffian duct. However, they exert opposing influences on the radial growth of the duct. As the epithelial cells within the Wolffian duct undergo cell division in random orientations within the tangential plane of the duct,^21,22^ cell proliferation contributes to the gradual expansion of the duct’s diameter over time. Conversely, mediolateral cell intercalation causes radial shrinkage of the duct as the duct extends.^28–30^ Interestingly, the radial size of the duct is largely maintained throughout the murine developmental process,^15,31^ indicating the presence of a regulatory multicellular system that balances the competing influences of cellular proliferation and intercalation to maintain the radial size of the developing duct.

It has been recently proposed that the cell intercalation counteracts mechanical forces associated with cell division to maintain the radial size of Wolffian duct.^22,32^ Live cell imaging has shown that mediolateral cell intercalation occurs along the circumferential axis of the duct in response to adjacent cell division. It is known that mitotic cell rounding occurs induced by hydrostatic pressure prior to cell division, building up pushing forces to the surrounding cells in densely packed epithelial tissues.^33,34^ As such, cell division along the circumferential axis of the duct transmits mechanical signals via compressive forces to neighboring cells, triggering active cell rearrangement in the neighboring cell clusters via myosin activation. This indicates the existence of an intrinsic mechanism through which cells sense mechanical forces specifically along the circumferential axis of the Wolffian duct. Furthermore, this represents a negative feedback control system, in which cells sense an increase in cell number along the circumference and corresponding changes in mechanical forces, leading to cell intercalation to regulate duct diameter. It is worth noting that individual constituent cells within the developing duct realize maintaining a consistent diameter throughout the duct by intrinsic force-mediated intercellular communications.

To achieve a comprehensive understanding of this system, future research is imperative in unraveling the molecular mechanisms that are responsible for sensing mechanical forces and transmitting signals to activate myosin. Molecular regulators of planar cell polarity, such as Vang-like proteins and Ptk7, which play a role in myosin phosphorylation,^35,36^ can be considered potential candidates that compose of a part of this system. Proteins of these regulators align circumferentially along the cellcell junctions of the ductal epithelial cells and have been demonstrated to cause the dilation of the developing duct when abrogated, because of disrupted mediolateral cell intercalation.^21,37^ Although significant efforts have been directed at explicating the molecular links between Ptk7 and Wnt signaling in broader contexts,^38–40^ the exact mechanism of anisotropic mechanosensing and transduction in the developing Wolffian duct is still unclear. It should be emphasized that mechanisms underlying radial size maintenance are not solely reliant on epithelial autonomy but also on other factors, including luminal fluid flow and smooth muscle contractility. Luminal flow, through mechanosensing ion channels, such as polycystin-1 (PC1) and polycystin-2 (PC2), plays a critical role in radial size regulation, with their disruption leading to epididymal duct dilation and subsequent failure of epididymal coiling.^41,42^ Another vital physical factor is the smooth muscle layers enveloping the Wolffian duct. Mesenchymal cells surrounding the duct differentiate into smooth muscle progenitors, gradually acquiring the potential to generate contractile forces radially to the duct during development from E16.5.^1,15^ It is reasonable to speculate that external contractile forces, induced by the smooth muscle progenitors, contribute to suppressing the radial enlargement of the Wolffian duct, in conjunction with the proposed mechanism during the late stage of epididymal morphogenesis.

## 3 | LUMINAL FLUID DYNAMICS IN MATURE EPIDIDYMIS

**FIGURE 2:**
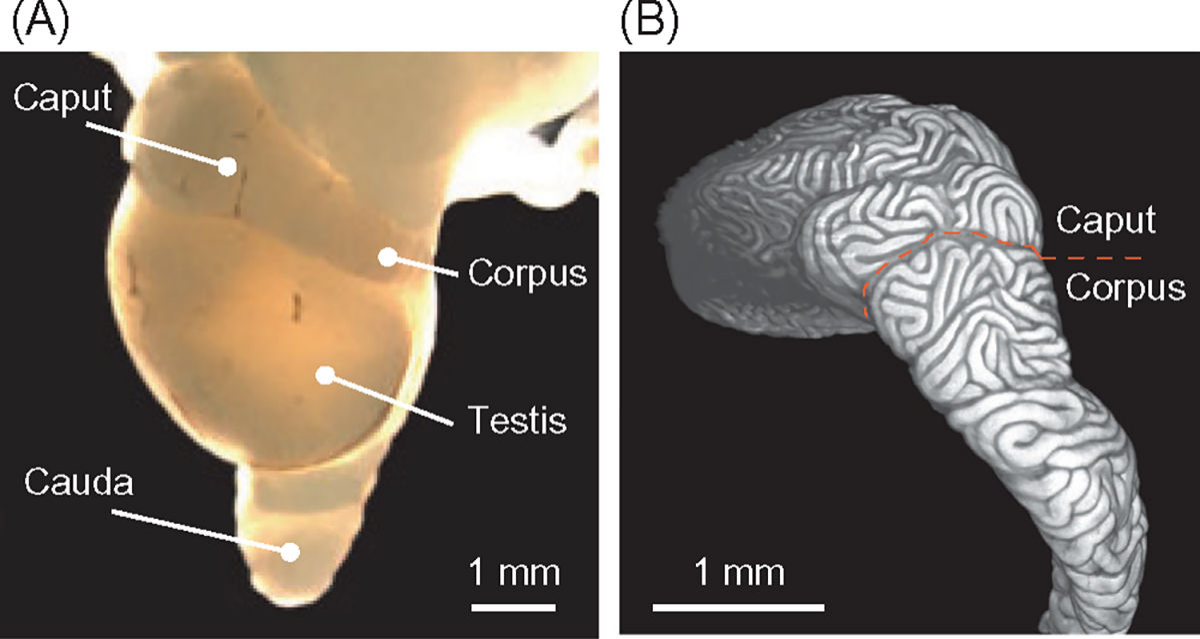
Structure of the adult murine epididymis. (A) Stereomicroscope image of the epididymis and testis. Scale bar, 1 mm. (B) Fluorescence image of the epididymal duct in the proximal region of epididymis. The boundary between caput and corpus is represented by the orange dotted line. Scale bar, 1 mm.

Once the mammalian epididymis has fully developed and matured, spermatozoa obtain their fertilization capacity while traversing through the elongated and convoluted epithelial duct (Figure 2). The movement of spermatozoa from the proximal (caput) to the distal (cauda) region of epididymis is mainly regulated by luminal fluid flow, which creates an environment where spermatozoa encounter and uptake luminal fluid that contains ions, organic solutes, and macromolecules secreted by the epithelial cells. This interaction results in the acquisition of motility and fertilization competency, referred to as sperm maturation. Recent genetic investigations have uncovered key genes and molecules that are expressed by the epididymal cells and are essential for the process of sperm maturation. However, the dynamic and mechanical aspects of the epididymal tissues that underlie the sperm maturation process remain incompletely understood. In this section, we discuss the generation of luminal fluid dynamics in the epididymis, the characterization of its dynamic properties, and epididymal mechanosensing and transduction in response to the luminal fluid flow.

### 3.1 | Origins of luminal flow

One of the major sources of long-term luminal flow of fluids throughout the epididymis is derived from the structural organization of the male reproductive system. Within the male mammalian reproductive system, the epididymal duct acts as a mediator between the seminiferous tubules, where spermatozoa are generated, and the urethra, where they are expelled. Spermatogonial stem cells located in the seminiferous tubules of the testis continuously give rise to spermatozoa after sexual maturation until death. Studies estimate that 4–8 × 10^7^ spermatozoa are produced per gram of testis in mice.^43,44^ Considering that the weight of the murine testis is approximately 0.1 g,^45^ roughly 5 million spermatozoa are produced per testis per day. As a significant number of spermatozoa are constantly produced at the closed-end of the male reproductive system, they are expelled from the other open-end of the tract. This source-sink relationship lays the basis of the unidirectional luminal flow throughout the epididymis on a day-to-week timescale.

Another critical factor contributing to the large-scale luminal flow within the epididymis is the result of luminal fluid secretion and absorption by the epididymal epithelial cells, which are crucial for establishing the luminal hydraulic environment.^46^ Principal cells, which are present throughout the entire epididymis, are considered primarily responsible for the secretion of fluids and chemicals into the lumen, particularly in the proximal regions of the epididymis, whereas they are involved in the reabsorption of various components from the epididymal fluids through endocytosis in more distal segments of the epididymis.^1,47^ The secreted components are believed to be the dominant constituents in the luminal fluid of the epididymis, as the majority of testicular fluid is reabsorbed before reaching the initial segments of the epididymis.^48,49^ Additionally, clear cells that are intermittently distributed throughout the epididymal epithelium are primarily responsible for the selective absorption of luminal components, as well as the maintenance of a luminal acidic environment.^1,46^ Recent efforts on understanding the mechanism of the luminal osmotic distribution have been insufficient despite of its importance for the resultant fluid flow within the epididymal duct.^50,51^ Interestingly, a study showed that the intraluminal hydrostatic pressure forms a gradient that increases from the caput to the cauda of the rat epididymis,^52^ indicating the necessity of other physical forces that propagate the flow of luminal fluid from the caput to the cauda.

Spontaneous contraction of the epididymal duct plays a vital role in the transportation of luminal flow, including spermatozoa, from the caput to the cauda, even when faced with a resting intraluminal hydrostatic pressure gradient. The smooth muscle cells surrounding the epithelial duct are responsible for the primary driving force behind the contraction of the epididymal duct, as previously described.^53,54^ It has been postulated that presumptive smooth muscle cells, established during the embryonic phase, undergo radial intercalation to form one to two layers of elongated, slender cells during the post-natal period. The thickness of the smooth muscle cells increases along the epididymal duct, from the caput to the cauda, and the contractile amplitude increases, whereas the frequency decreases toward the cauda.^54–57^ Although hormonal and signaling effects have been studied to some extent,^53,58–60^ the spatiotemporal regulation of ductal contraction in physiological conditions remains unknown. The organization of the spatiotemporal pattern of epididymal ductal contractions is crucial for the forward movement of luminal fluidic contents. Local contraction of the duct creates a region of higher pressure and lower volume, leading to a pressure gradient that causes luminal fluid to move from the region of higher pressure toward the area of lower pressure. When the contracted region of the duct relaxes, the pressure inside equalizes, causing the fluid to move back toward its original position. Depending on the properties of the fluid and the duct, this movement may result in oscillations before the fluid reaches its original position within a short time frame. If the contractions were coordinated in a peristaltic manner, the luminal flow would be able to move toward the distal side. One potential factor involved in coordinating smooth muscle contractions is the interstitial cells of Cajal, known as the pacemaker for contraction.^58,61,62^ The interplay between smooth muscle cells and interstitial cells of Cajal in the epididymis is not yet fully understood. Further investigation is required to clarify epididymal luminal flow triggered by ductal contraction.

The movement of the sperm flagellum may induce fluid flow in microenvironments of the epididymal duct following the acquisition of sperm motility during the passage along the duct. An in vitro assay using murine epididymis demonstrated that the spermatozoa exhibit irregular motions with limited forward movement in the caput region, but the movement is significantly enhanced as the spermatozoa reach the middle corpus region,^63^ suggesting that the self-propelling capacity of spermatozoa may be endowed somewhere between the caput and middle corpus. The collective dynamics of sperm flagellar beating can trigger collective sperm behaviors and generate distinct luminal fluid through hydrodynamic and physical interactions with material properties and geometric constraints of the epididymal environment.^64,65^ Despite the self-propelling capacity of spermatozoa, in vitro assays have indicated that they are stored quietly in the cauda of the epididymis.^66,67^ Further investigation is required to clarify sperm-induced luminal flow in physiologically relevant conditions.

### 3.2 | Speed and type of luminal sperm flow

Here we aim to estimate the average flow speed of luminal spermatozoa throughout the entire epididymis. The primary objective of this section is to perform a back-of-the-envelope calculation to gain insight into the rough order of the luminal sperm flow rate. Another objective is to summarize measured data on the total length along the epididymal duct and sperm transit time within the entire epididymis obtained from previous studies, which are both necessary for estimating the luminal sperm flow rate.

Measuring the length of the epididymal duct is challenging because of its complex and irregular three-dimensional structure. Furthermore, there are no uniform folding patterns across different regions of the epididymis. The standard method for morphological quantification of the epididymis is based upon a stereological approach, which estimates three-dimensional parameters, such as the total longitudinal length and volume of the epididymal duct, from two-dimensional section images. To achieve the most accurate results within this framework, serial sections with reconstruction through image analysis are recommended. For example, the analysis of a reconstructed 3D structure of the murine epididymis estimated its entire length to be approximately 0.7–0.8 m.^26^ It should be noted that the accuracy of stereological inference depends on the quality of the tissue sample and the precision of the measurements, which can result in large variances of data. Studies have demonstrated significant variation in the estimated longitudinal length of rat epididymal duct. In the case of the rat epididymis, there have been nearly fourfold differences in the estimated longitudinal length, ranging among ~6.5,^68^ ~3.2,^54^ and ~1.5 m.^69^ Similarly, studies on the human epididymis reported the length of the epididymal duct as ranging between 3 and 6 m.^70,71^ These variations in measurement are because of inconsistencies in measurement methods and a lack of direct measurement. Hence, direct measurement using whole-epididymal optical clearing and imaging is necessary to obtain more precise morphological information.^72,73^

Given that the transit time of spermatozoa in the murine epididymis is approximately 10 days,^1,74^ the average speed of luminal sperm flow in the murine epididymis is estimated to be 53 *μ*m min^−1^, which corresponds to roughly half of the total sperm length per minute.^75,76^ In the case of rats, the sperm transit time through the epididymis is approximately 13 days^54,77^ and assuming the length of the rat epididymal duct would be 3 m, then the average speed of luminal sperm flow is approximately 160 *μ*m min^−1^. Because rat sperm length is about 160 *μ*m,^78^ spermatozoa can move forward one sperm length every minute in the epididymis. These rough calculations suggest that there is no significant difference between these rodents in the average speed of the relative luminal sperm flow to the sperm length.

**FIGURE 3:**
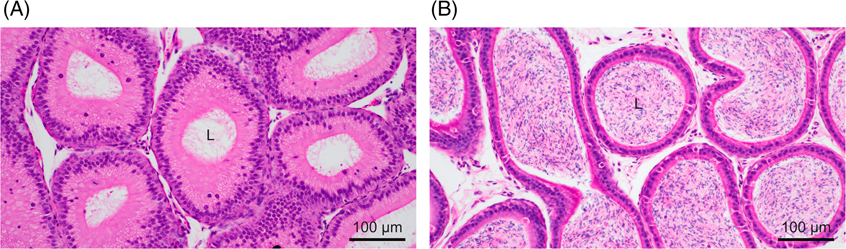
Section images of the murine epididymis. Paraffin slices stained with hematoxylin and eosin in the initial segment (A) and the cauda (B). No spermatozoa can be observed in the lumen in the initial segment in contrast to the cauda. Scale bars, 100 *μ*m. L, lumen of the duct.

It should be noted that the luminal sperm flow rate is not constant and is highly variable across different regions of the epididymis. For example, a study in anesthetized rats that injected oil droplets into various regions of the epididymal duct found that the transport net speed and dispersion of the droplets within a fixed time were greater in the proximal regions compared to the distal region.^55^ The flow rate of spermatozoa is expected to be remarkably higher in the initial segment compared to other regions, which is supported by the infrequent presence of spermatozoa in the epididymal lumen in this particular segment (Figure 3). In addition, a detailed analysis revealed that oil droplets exhibited several types of movements in the cauda region of rat epididymis, including bidirectional movements leading to little or large net forward movement and relatively straightforward unidirectional movement in an observation time window.^54^

Despite these detailed investigations, our understanding of luminal sperm flow in the epididymis remains incomplete, as there are no reports on the observation of sperm dynamics at the single cell resolution under physiological conditions. To gain a more thorough understanding of luminal sperm flow in the epididymis under physiological conditions, a promising approach is intravital imaging of live animals using two-photon excitation microscopy (TPEM).^79,80^ This technique offers the benefits of low phototoxicity and high penetration depth in light-scattering samples in live animals. For example, intravital imaging of murine testis and sperm cells using TPEM has revealed that the orientation of sperm flagella intermittently reverses over time, possibly caused by testicular luminal flow in live mice.^81^ TPEM also provides label-free imaging techniques, such as second-harmonic generation, which can capture collagen fibers during acute erectile responses of murine genital tissues.^82^ As imaging techniques continue to advance, a more comprehensive characterization of sperm collective dynamics in the epididymis can be achieved under physiological conditions.

### 3.3 | Flow-induced mechano-sensation and transduction in epididymis

Luminal fluid flow in the epididymis generates mechanical forces, which are applied to the duct epithelial cells through compression and shear stress. These forces may play a role in triggering cellular responses in the tissues that come into contact with the luminal fluids via mechanosensing and signal transduction pathways.^83,84^ Similar mechanisms have been identified in other tissues, such as endothelial cells in blood vessels and osteocytes in bones.^85–88^ However, specific effects of these mechanical forces on the epididymal epithelium and the underlying mechanotransduction pathways remain largely unexplored.

Mechanosensitive ion channels are specialized membrane proteins that can detect alterations in plasma membrane tension or distortion induced by fluid shear stress and activate by opening to facilitate ion transport across the membrane. These channels play a pivotal role in many cell types, with Piezo1 and Trpv4 being the key molecules involved.^89–92^ Recently, the presence of Piezo1 channels has been identified in the rat epididymal epithelium, with activated Piezo1 channels enabling Ca^2+^ influx into epithelial cells and activating large conductance Ca^2+^-activated K^+^ (BK_Ca_) channels for transepithelial K^+^ secretion.^93^ This ion flow can trigger a cascade of downstream signaling events that ultimately lead to cellular responses. Additionally, Trpv4 channels, known as a shear stress sensor,^90,94^ are involved in transepithelial K^+^ secretion in the epididymis.^95,96^ These recent discoveries suggest that PIEZO1 and TRPV4 may have a critical role in mechanosensing fluid shear stress and regulating the physiological maintenance of the luminal environment in the epididymal duct. However, the exact mechanisms by which these channels respond to mechanical stimuli and control fertility remain unclear. Future studies employing tissue-specific knockout strategies may provide insights into the physiological functions of these mechanosensors in the epididymis.

Primary cilia have been demonstrated to detect extracellular fluid flow and elicit intracellular responses across various tissues.^97,98^ The immotile primary cilia structure is characterized by an axoneme with nine microtubule doublets that are arranged in a circle without central microtubules, a configuration commonly referred to as the 9 + 0 structure. This specific architecture allows for primary cilia to have a flexible structure and respond to mechanical stimuli through bending. The flow-induced bending of primary cilia causes the displacement of the ciliary membrane, subsequently leading to the opening of ion channels such as PC2 or its encoding gene *Pkd2*, which are located on the membrane.^99–101^ Activation of stretch-activated channels PC2 enables ion influx, such as Ca^2+^ into the cytoplasm, leading to downstream signaling pathways that mediate cellular responses to mechanical stimuli.^102,103^ Interestingly, PC2 and TRPV4 interact and colocalize in primary cilia to form a mechanosensitive molecular sensor.^104^ In the murine and equine epididymis, primary cilia are found in undifferentiated columnar cells from birth to puberty and in basal cells and peritubular myoid cells in adulthood.^105,106^ Similar findings were reported in the equine epididymis as well.^107^ The mechanosensitive ion channel PC2 is localized on the cilia of DC2 cells, the immortalized mouse epididymal epithelial cell line isolated from the distal caput,^105,108^ suggesting that the primary cilia of the epididymal epithelium may function as a mechanosensing unit for detecting luminal fluid flow prior to puberty.

Aside from primary cilia, stereocilia may also serve as additional possible mechanosensors of luminal fluid flow in the epididymis. Stereocilia, present in epididymal principal cells, are composed of bundles of actin filaments cross-linked by various proteins, providing structural integrity. At the tip of each immotile stereocilium lies, an actin-rich structure called the tip link, which connects the tips of adjacent stereocilia and is essential for mechanosensing and transduction.^109–111^ When stereocilia are deflected by fluid movement, the tip links stretch, and ion channels located at the tips of the stereocilia open, triggering Ca^2+^ entry into the cells.^112–114^ Although stereocilia have been observed to project from epididymal principal cells, their function has been limited to absorption of the luminal fluids.^115–117^ Investigating the response of epididymal stereocilia to luminal flow could lead to the discovery of unexplored mechanotransduction mechanisms.

## Data Availability

The data that support the findings of this study are available from the corresponding author upon reasonable request.
